# The Association of Serum 25-Hydroxyvitamin D Concentrations and Elevated Glycated Hemoglobin Values: A Longitudinal Study of Non-Diabetic Participants of a Preventive Health Program

**DOI:** 10.3390/nu9070640

**Published:** 2017-06-22

**Authors:** Lalani L. Munasinghe, Marco F. Mastroeni, Silmara S. B. S. Mastroeni, Sarah A. Loehr, John Paul Ekwaru, Paul J. Veugelers

**Affiliations:** 1 Population Health Intervention Research Unit, School of Public Health, University of Alberta, 3-50 University Terrace, 8303–112 St, Edmonton, AB T6G 2T4, Canada; lalani.m@ualberta.ca (L.L.M.); marco.mastroeni@univille.br (M.F.M.); silmara.mastroeni@univille.br (S.S.B.S.M.); Sarah.Loehr@ualberta.ca (S.A.L.); ekwaru@ualberta.ca (J.P.E.); 2 Post-Graduation Program in Health and Environment, University of Joinville Region, Rua Paulo Malschitzki, n 10, Joinville 89.219-710, Brazil; 3 Department of Physical Education, University of Joinville Region, Rua Paulo Malschitzki, n 10, Joinville 89.219-710, Brazil

**Keywords:** serum 25-hydroxyvitamin D, vitamin D, glycated hemoglobin, hemoglobin A_1c_, diabetes, prevention

## Abstract

The prevalence of Type 2 Diabetes (T2D) is sharply on the rise, both in Canada and worldwide. As addressing its root causes, i.e., promotion of healthy lifestyles and weight management, has been largely unsuccessful, new clues for primary prevention seem essential to curbing the increasing public health burden of T2D. In the present study, we examined whether improvements in vitamin D status, i.e., serum 25-hydroxyvitamin D [25(OH)D] concentrations, are paralleled by a reduction in the risk for reaching adverse glycated hemoglobin (HbA_1c_) levels in a community sample of non-diabetic volunteers participating in a preventive health program that encourages the use of vitamin D. Repeated observations on 6565 participants revealed that serum 25(OH)D concentrations increased from 90.8 to 121.3 nmol/L, HbA_1c_ values decreased from 5.6% to 5.5%, and the prevalence of having HbA_1c_ values ≥ 5.8% decreased from 29.5% to 17.4% while in the program. Compared to participants who did not increase their 25(OH)D concentrations during follow-up, those who increased their 25(OH)D concentrations with 50 nmol/L or more were 0.74 times as likely to achieve elevated HbA_1c_ values at follow-up (*p* = 0.03). These findings suggest that public health initiatives that promote vitamin D status along with healthy lifestyles in the population at large may alleviate the future public health burden associated with T2D.

## 1. Introduction

The prevalence of diagnosed Type 2 Diabetes (T2D) in Canada is sharply on the rise. It reached 9.3% in 2015 and is projected to reach 13.4% and to cost $14.6 billion in direct and indirect healthcare expenditures in 2025 [1]. As a chronic metabolic disorder resulting from insufficient or ineffective insulin to control blood glucose concentration [2,3], T2D is primarily attributable to poor lifestyles and excess body weight. Promotion of healthy lifestyles and weight management, unfortunately, has been unsuccessful in curbing the increasing public health burden of T2D.

Vitamin D is a steroid hormone synthesized endogenously when the skin is exposed to ultraviolet rays from sunlight [4,5]. Individuals without sufficient exposure to sunlight, such as Canadians who live at northern latitudes, rely on vitamin D obtained through diet and supplementation [4,5]. The role of vitamin D in maintaining bone health has been well established [4,6], however, ubiquitous vitamin D receptors expressed in diverse cells throughout the body imply that vitamin D plays various other roles in metabolism and health. Vitamin D has been suggested to be involved in the pathogenesis of T2D, though the mechanism remains unclear. It has been suggested to have direct effects on insulin secretion [5,7,8,9], insulin resistance [5] and sensitivity [7], as well as on insulin action [9]. Any of these effects would achieve a lowering in glycated hemoglobin or hemoglobin A_1c_ (HbA_1c_), which measures the three-month average plasma glucose concentration. HbA_1c_ has been established as a screening test for T2D [10,11,12] and an indicator for glycemic control for patients with T2D [12,13,14]. The suggested role of vitamin D in the pathogenesis of T2D had prompted various studies involving HbA_1c_. Cross-sectional studies overwhelmingly demonstrated the existence of an inverse association of circulating 25-hydroxyvitamin D [25(OH)D], the nutritional biomarker for vitamin D status, with HbA_1c_ concentration, both in patient groups [15,16,17,18,19,20,21] and community samples [17,22,23], though a few studies failed to confirm this association [13,24]. Randomized controlled trials (RTCs) studying the effect of vitamin D supplementation regimens have been less consistent, with the majority not showing a lowering in HbA_1c_ values [7,8,25,26,27,28,29,30,31,32] and a minority showing a reduction in HbA_1c_ concentration [33,34,35]. These RCTs typically focused on patient groups with T2D or at risk for T2D. The RCTs typically lasted 3 or 4 months, the lifespan of red blood cells [36], to test the hypothesis that HbA_1c_ values would come down. To the best of our knowledge, no intervention studies in community samples have examined the potential of vitamin D to slow down the progression to T2D among community members, whereas this seems essential to public health decision makers in charge of articulating primary preventions strategies in their attempts to curb the public health burden of T2D. Such studies typically require longer follow-up and larger sample sizes. In the present study, we examine whether temporal increases in serum 25(OH)D concentrations are paralleled by a reduction in the risk for reaching adverse HbA_1c_ concentrations in a community sample of non-diabetic volunteers participating in a preventive health program that encourages the use of vitamin D supplements.

## 2. Methods

### 2.1. Study Design and Participants

The present study analyzed data from the Pure North S’Energy Foundation (PN), a not-for-profit organization, in Calgary, Alberta, Canada that offers a preventative health program to community volunteers. Details of the program have been previously published elsewhere [37]. Information can be found on the PN website [38]. Briefly, PN was established in 2007 and employs health professionals who offer lifestyle counselling to volunteer participants. At enrolment, participants complete a demographic questionnaire and provide information regarding their medical history and the use of medication. At enrolment and follow-up visits, participants complete lifestyle questionnaires, have their weight and height assessed, blood pressure measured, and blood drawn for the assessment of several biomarkers including serum 25(OH)D concentrations and HbA_1c_ values. This information is used to guide the lifestyle counselling. Vitamin D supplementation is encouraged, given that Canada’s northern latitude limits sunlight exposure, resulting in inadequate cutaneous synthesis of vitamin D. Follow-up visits for both assessments and counselling are scheduled annually. Participants granted written informed consent for the use of their information for research purposes. All data were anonymized by PN prior to it being transferred to the University of Alberta for data analyses. The Human Research Ethics Board at the University of Alberta provided the ethical approval for the analyses of the PN data.

Data were obtained from PN for 8007 adult participants who enrolled in the program between October 2007 and April 2014 and had at least two measures of both serum 25(OH)D concentrations and HbA_1c_ values. Where assessments of more than one follow-up visits were available, the present study used the endmost visit. This study focused on non-diabetic individuals, and therefore, those who were having physician’s diagnosed diabetes/taking medication for diabetes (*n* = 59) or having undiagnosed diabetes, i.e., those with HbA_1c_ values ≥6.5% (*n* = 1204) or with fasting blood glucose concentration ≥7 mmol/L (*n* = 53) at the baseline, were excluded [11,39,40]. An additional 126 participants were excluded for whom the time between baseline and follow-up was less than 3 months. The analysis included the data of 6565 participants.

### 2.2. HbA_1c_ Values

HbA_1c_ concentrations in blood were assessed using the Beckman Coulter^®^ AU680 (Beckman Coulter, CA, USA), which has an inter-lab correlation coefficient of 0.96 (*n* = 242, *p* < 0.001). HbA_1c_ values were expressed in percent (%) according to Diabetes Control and Complications Trial (DCCT) units. HbA_1c_ values were categorized into two groups as “low diabetes-risk (<5.8%)” and “increased diabetes-risk (≥5.8%)” [36], while recognizing alternative cut-off points have also been recommended and applied [39,40,41].

### 2.3. Serum 25(OH)D Concentrations

DiaSorin Liason^®^ (DiaSorin S.p.A., Saluggia (VC), Italy) assessed serum 25(OH)D concentrations using chemiluminescent immunoassay with an inter-assay coefficient of variation (CV) of 11%. Baseline concentrations were categorized into five groups (<50, 50 to <75, 75 to <100, 100 to <125, and ≥125 nmol/L). Change in 25(OH)D was calculated by subtracting the baseline concentration from the follow-up concentration and then categorized into five groups (no improvement, increased by <25, increased by 25 to <50, increased by 50 to <75, and increased by ≥75 nmol/L).

### 2.4. Other Covariates

The concentrations of serum triglycerides (inter-assay CV = 2%), total cholesterol (inter-assay CV = 1.5%), and high-density lipoprotein (HDL; inter-assay CV = 2%) were measured using Automated Cobas^®^ 8000 Modular Analyzer Series. Low-density lipoprotein (LDL) concentrations were calculated using the formula, “LDL = Total Cholesterol − HDL − (Triglycerides/2.2)”, and categorized as “Normal (<2.6 mmol/L)” and “Elevated (≥2.6 mmol/L)”. “Normal blood pressure” was defined as systolic and diastolic pressures <140/90 mmHg, and “Elevated blood pressure” as blood pressures ≥140/90 mmHg [42] or for those using anti-hypertensive medication at baseline. As none of the eligible study participants had iron deficiency anemia based on serum ferritin and serum iron concentrations [43] and none received medication for renal failure or had an estimated glomerular filtration rate <15 mL/min/1.73 m^2^ [44], adjustment for iron deficiency and renal function were not applied. Self-reported lifestyle questionnaires provided information on age, gender, smoking status (“never smoker”, “past smoker”, and “current smoker”), alcohol consumption (“non-drinker”, “drinker”) and physical activities for a typical week (light, moderate, and vigorous activities). “Non-drinker” was defined as one who reported drinking <2 glasses per week and ‘drinker” as one who drank ≥2 glasses per week. The metabolic equivalent of task (MET) per week was obtained from each physical activity recorded in the questionnaire and then was multiplied by the time participants reported performing those activities per week (MET × hours per week). The total MET hours for each week were categorized as “low (<10 MET hours/week)”, “moderate (10 to <20 MET hours/week)”, and “high (≥20 MET hours/week)”. Body mass index (BMI) was calculated as weight in kg/height^2^ (kg/m^2^) and was then categorized as “underweight (<18.5 kg/m^2^)”, “normal weight (18.5 to <25 kg/m^2^)”, “overweight (25 to <30 kg/m^2^)”, and “obese (≥30 kg/m^2^)” [45]. Underweight and normal weight individuals were combined into one group in the regression analysis due to the low prevalence of underweight.

### 2.5. Statistical Analyses

Descriptive statistics included percentages, medians with interquartile ranges (IQR), and means with standard deviation (SD). Differences in median, mean, and frequencies of categorical variables between baseline and follow-up were compared using the Wilcoxon matched-pairs signed-ranks test, the Paired *t*-test, and the McNemar’s chi-square test, respectively. Cross-sectional associations of serum 25(OH)D concentrations with HbA_1c_ values were examined for baseline observations using linear regression models and expressed in change in HbA_1c_ concentration per 25 nmol/L change in 25(OH)D concentration. Multiple logistic regression was applied to quantify the longitudinal relationships of 25(OH)D at baseline and changes in 25(OH)D during follow-up with elevated HbA_1c_ at follow-up. These regression analyses were adjusted for elevated HbA_1c_ at baseline and for age, gender, and baseline values of LDL-cholesterol, blood pressure, smoking status, body weight, alcohol consumption, and physical activity level. Sub-analyses would further consider the potential influence of LDL-cholesterol, blood pressure, smoking status, body weight, alcohol consumption, and physical activity level at follow-up. Missing data for covariates were treated as a separate category in these regression analyses. All statistical analyses were performed using Stata, version 14.0 (Stata Corp., College Station, TX, USA) with statistical significance at 0.05.

## 3. Results

Table 1 depicts the characteristics of the 6565 participants at the baseline and at follow-up. The median follow-up was 1.1 years (IQR = 0.9–1.6 years). The mean serum 25(OH)D concentrations increased from 90.8 nmol/L at baseline to 121.3 nmol/L at follow-up, while the mean HbA_1c_ values decreased from 5.6% at baseline to 5.5% at follow-up. The prevalence of participants with increased diabetes-risk, i.e., those with HbA_1c_ values ≥ 5.8%, decreased from 29.5% to 17.4% (Table 1).

Linear regression of baseline observations revealed that for every 25 nmol/L increment in 25(OH)D concentrations, HbA_1c_ values appeared 0.008% (as a percentage point difference) lower (95% confidence interval (CI: −0.013% to −0.004%; *p* ≤ 0.01). When this cross-sectional association was adjusted for age, gender, LDL-cholesterol, blood pressure, body weight status, smoking status, alcohol consumption, and physical activity, HbA_1c_ values were 0.007% (CI: −0.012% to −0.002%; *p* = 0.01) lower for every 25 nmol/L increment in 25(OH)D concentrations.

Table 2 depicts the results of the longitudinal data analysis. Subjects who increased their 25(OH)D concentrations by 50 to 75 nmol/L or by more than 75 nmol/L were 0.74 times as likely to achieve elevated HbA_1c_ values at follow-up compared to those who did not increased their 25(OH)D concentrations during follow-up. Also, subjects with baseline 25(OH)D concentrations above 100 nmol/L were statistically significantly less likely to achieve elevated HbA_1c_ values compared to those who had 25(OH)D concentrations below 50 nmol/L. Furthermore, older age, male gender, elevated LDL-cholesterol, excess body weight, and elevated blood pressure increased the risk for elevated HbA_1c_ at follow-up, i.e., after a median duration of 1.1 years. Sub-analyses that further considered the potential effects of LDL-cholesterol, blood pressure, smoking status, body weight, alcohol consumption, and physical activity at follow-up did not reveal a statistical significant role in addition to their baseline values, nor did they affect the estimates of the effect of baseline 25(OH)D and of changes in 25(OH)D on HbA_1c_ values in a meaningful manner.

## 4. Discussion

We revealed that for a large group of non-diabetic participants enrolled in a program that encourages vitamin D supplements that, on average, HbA_1c_ values declined. Those participants with above average vitamin D status, i.e., higher baseline 25(OH)D concentrations, and those who increased their 25(OH)D concentrations during follow-up were less likely to progress to HbA_1c_ values in excess of 5.8%, the cutoff for increased risk for T2D, relative to those with lower baseline 25(OH)D concentrations or without increases in 25(OH)D concentrations during follow-up.

The decline in HbA_1c_ values, from 5.6% at baseline to 5.5% at follow-up, is seemingly modest. A review of the literature reported that a reduction of 0.1% and 0.2% in HbA_1c_ values was reflected in a decrease of 6% and 13% in the prevalence of self-reported diabetes, respectively [12]. The observed decline represents an average of the 6565 participants. It is important to note that not all participants improved their 25(OH)D concentrations. Specifically, 1523 (23%) participants did not improve their 25(OH)D concentrations during follow-up. Relative to this subgroup, those who did improve their 25(OH)D concentrations were substantially less likely to become at risk for T2D (reaching HbA_1c_ values in excess of 5.8% [36]). This observation seems to contradict the majority of RCTs concluding no effect of vitamin D supplementation on lowering HbA_1c_ values [7,8,25,26,27,28,29,30,31,32] and to confirm the fewer RCTs concluding the existence of such an effect [33,34,35]. There are, however, important differences between previous RCTs and the present study. The present study was conducted in a community sample of volunteer participants free of T2D, whereas almost all previous RCTs had been conducted among patients with T2D or at risk for T2D. This was reflected in higher baseline HbA_1c_ values reported in those RCTs than the 5.6% observed in the present study. One may speculate that vitamin D is more effective at slowing progression when HbA_1c_ values are still low rather than reversing HbA_1c_ values once these have reached higher levels. A second important difference is that the present study examined changes in 25(OH)D concentrations, whereas previous RCTs examined the effects of vitamin D supplementation regimens. Changes in 25(OH)D concentrations not only capture the effect of vitamin D supplementation but also the potential contributions of sunlight and vitamin D in the diet. Changes in 25(OH)D concentrations are absolute, whereas responses to vitamin D regimens vary considerably and differ by body weight [37,46]. For example, relative to normal weight subjects, obese individuals need 2 to 3 times more vitamin D to achieve the same 25(OH)D concentration. The third important difference is that unlike previous RCTs the present study is without randomization and blinding. In fact, participants’ vitamin D status is often the basis for the vitamin D supplementation dose that is being recommended. As such, the present study is more prone to bias, though does represent a real-world approach to optimizing vitamin D status.

We observed that HbA_1c_ values were 0.007% lower for every 25 nmol/L increment in 25(OH)D concentrations. This is consistent with the inverse association between 25(OH)D and HbA_1c_ values reported for non-diabetics in the National Health and Nutrition Examination Survey [23] and various other cross-sectional comparisons in community samples [16,17,22] and patient groups [15,17,18,19,20,21]. We also identified older age, male gender, elevated LDL-cholesterol, excess body weight, and elevated blood pressure as risk factors for progression to adverse HbA_1c_ values. These are the established risk factors for diabetes [2,3] and for cardiovascular disease [47,48]. A recent study [49] identified an inverse association of alcohol consumption with HbA_1c_. However, in the present study, we observed this association to not be statistically significant. Earlier, we had shown that an adequate vitamin D status along with physical activity and markers of a healthy lifestyle (body weight, LDL cholesterol, blood pressure) lowered the risk of metabolic syndrome [50] and insulin resistance [51] in this population. Where promotion of healthy eating and physical activity as well as weight management is currently the established approach to reduce the risk for T2D and other chronic diseases, the present study makes the case to complement this approach with promotion of maintaining an adequate vitamin D status.

Strengths of the present study are its longitudinal design, large sample size, and broad range of serum 25(OH)D concentrations. An additional strength of the study is the fact that we were able to adjust for a series of potential confounding covariates. We acknowledge that this study had a study limitation in that it was conducted among subjects who volunteered to participate in the preventive health program. These participants are likely health conscious and motivated to comply with the lifestyle advise the program provides to them. These participants are therefore not representative of the general population. The preventive health program not only encourages supplementation with vitamin D but also healthy lifestyles in general. In this regard, we previously reported that physical activity levels improved during follow-up [52], however, our sub-analyses showed that physical activity at follow-up did not affect the estimates of the effect of baseline 25(OH)D and of changes in 25(OH)D on HbA_1c_ values in a meaningful manner. Other lifestyle changes may also have occurred, though were not recorded. However, our sub-analyses showed that markers of a healthy lifestyle (body weight, LDL cholesterol, blood pressure) at follow-up did not affect the estimates for baseline 25(OH)D and changes in 25(OH)D on HbA_1c_ values either. The existence of missing information for the various confounding variables (detailed in Table 1) may have contributed to an incomplete accounting of their confounding potential. We did, however, conduct a series of sub-analyses restricted to participants with complete information that revealed very similar results as the ones presented in Table 2. The various limitations underline that caution is warranted in the generalization of the present findings.

In conclusion, the present study revealed that improving the vitamin D status of community members reduces the likelihood of becoming at risk for T2D. Public health initiatives that promote vitamin D status along with healthy lifestyles in the general public may alleviate the future public health burden associated with T2D. More longitudinal studies in healthy subjects are suggested to further elucidate the potential of vitamin D to slow progression to T2D.

## Figures and Tables

**Table 1:** Baseline and follow-up characteristics of 6565 study participants.

	Baseline	Last Follow-Up	*p*-value ^§^
**Serum 25(OH)D, nmol/L**			
Mean (SD)	90.8 (41.5)	121.3 (46.5)	
Median (IQR)	84.0 (63.3–110.0)	115.0 (88.6–147.0)	<0.01
**HbA_1c_ value, %**			
Mean (SD)	5.6 (0.3)	5.5 (0.3)	<0.01
Median (IQR)	5.6 (5.4–5.8)	5.4 (5.2–5.7)	<0.01
**Elevated HbA_1c_ value, %**			
Increasing diabetes risk (≥5.8%)	1937 (29.5)	1145 (17.4)	<0.01
Low diabetes risk (<5.8%)	4628 (70.5)	5420 (82.6)	
**Age, mean (SD)**	50.6 (15.0)	52.1 (14.9)	<0.01
**Gender (%)**			-
Female	3510 (53.5)	3510 (53.5)	
Male	3055 (46.5)	3055 (46.5)	
**Body weight status, %**			0.79
Underweight	77 (1.2)	75 (1.2)	
Normal weight	2295 (35.4)	2290 (35.3)	
Overweight	2467 (38.0)	2437 (37.6)	
Obese	1647 (25.4)	1684 (26.0)	
Missing	79	79	
**Blood pressure, % ^†^**			<0.01
Normal	5660 (90.5)	5291 (91.6)	
Elevated	596 (9.5)	486 (8.4)	
Missing	309	788	
**Serum LDL-cholesterol, % ^‡^**			<0.01
Normal	2222 (35.3)	2022 (31.5)	
Elevated	4077 (64.7)	4389 (68.5)	
Missing	266	154	
**Smoking status, %**			0.02
Never smoker	2726 (56.2)	1983 (57.1)	
Past smoker	1477 (30.4)	1019 (29.3)	
Current smoker	649 (13.4)	472 (13.6)	
Missing	1713	3091	
**Alcohol consumption status, %**			<0.01
Non-drinker	2069 (44.4)	2136 (46.9)	
Drinker	2588 (55.6)	2414 (53.1)	
Missing	1908	2015	
**Physical activity level, %**			<0.01
Low	1930 (39.6)	1748 (36.3)	
Moderate	1504 (30.8)	1510 (31.4)	
High	1441 (29.6)	1556 (32.3)	
Missing	1690	1751	
**Use of vitamin D-containing supplements, %**			<0.01
Yes	3436 (58.1)	4901 (90.4)	
No	2474 (41.9)	519 (9.6)	
Missing	655	1145	
**Vitamin D intake from supplements among users, IU/day**			
Mean (SD)	3866.7 (3628.6)	7256.8 (3543.4)	
Median (IQR)	3000 (2000–5000)	7000 (5000–10,000)	<0.01

Abbreviations: 25(OH)D, 25-hydroxyvitamin D; LDL-cholesterol, low-density lipoprotein cholesterol; SD, standard deviation; IQR, interquartile range. ^§^ *p*-values: Differences in median, mean, and frequencies of categorical variable between baseline and follow-up were compared using the Wilcoxon matched-pairs signed-ranks test, the Paired *t*-test, and the McNemar’s chi-square test, respectively; *p*-values for differences in means are presented only for those variables with normal distributions. **^†^** Blood pressure status was defined based on blood pressure ≥140/90 mmHg, or a self-report of taking antihypertensive medications as elevated. **^‡^** Elevated LDL-cholesterol was defined as LDL-cholesterol concentration ≥2.6 mmol/L.

**Table 2:** Risk for elevated glycated hemoglobin concentration (≥5.8%) at last follow-up among 6565 participants.

	# of Participants	Univariable OR (95% CI)	*p*-value	Multivariable ^§^ OR (95% CI)	*p*-value
**Serum 25(OHD) at baseline, nmol/L**					
<50	780	reference		reference	
50–<75	1784	0.81 (0.66, 0.99)	0.04	0.84 (0.66, 1.07)	0.17
75–<100	1824	0.68 (0.55, 0.83)	<0.01	0.80 (0.63, 1.03)	0.09
100–<125	1140	0.50 (0.39, 0.63)	<0.01	0.57 (0.43, 0.76)	<0.01
≥125	1037	0.43 (0.34, 0.56)	<0.01	0.51 (0.37, 0.71)	<0.01
**Change in serum 25(OH)D, nmol/L**					
No improvement	1523	reference		reference	
Increase of <25	1601	1.19 (0.98, 1.44)	0.07	0.90 (0.71, 1.13)	0.36
Increase of 25–<50	1468	1.25 (1.03, 1.51)	0.02	0.84 (0.66, 1.07)	0.15
Increase of 50–<75	974	1.16 (0.94, 1.44)	0.17	0.74 (0.56, 0.97)	0.03
Increase of ≥75	999	1.14 (0.92, 1.41)	0.24	0.74 (0.57, 0.97)	0.03
**Serum HbA1c ≥5.8% at baseline**					
No	4628	reference		reference	
Yes	1937	11.04 (9.53, 12.79)	<0.01	10.72 (9.09, 12.63)	<0.01
**Serum LDL-cholesterol at baseline ^†^**					
Normal	2222	reference		reference	
Elevated	4077	1.40 (1.22, 1.62)	<0.01	1.20 (1.01, 1.41)	0.03
**Age at baseline (per 10 years)**	6565	1.31 (1.25, 1.37)	<0.01	1.18 (1.11, 1.26)	<0.01
Gender					
Female	3510	reference		reference	
Male	3055	1.46 (1.28, 1.66)	<0.01	1.51 (1.29, 1.77)	<0.01
**Body weight status at baseline**					
Underweight/normal weight	2372	reference		reference	
Overweight	2467	1.75 (1.48, 2.08)	<0.01	1.40 (1.15, 1.70)	<0.01
Obese	1647	1.56 (3.00, 4.22)	<0.01	2.15 (1.76, 2.63)	<0.01
**Blood pressure status at baseline ^‡^**					
Normal	5657	reference		reference	
Elevated	849	1.96 (1.65, 2.31)	<0.01	1.27 (1.04, 1.55)	0.02
**Smoking status at baseline**					
Never smoker	2726	reference		reference	
Past smoker	1477	1.08 (0.91, 1.28)	0.39	0.97 (0.79, 1.18)	0.75
Current smoker	649	1.15 (0.92, 1.45)	0.22	1.16 (0.89, 1.53)	0.28
**Alcohol consumption status at baseline**					
Non-drinker	2069	reference		reference	
Drinker	2588	0.74 (0.63, 0.87)	<0.01	0.84 (0.69, 1.01)	0.06
**Physical activity level at baseline**					
Low	1930	reference		reference	
Moderate	1504	0.77 (0.64, 0.92)	<0.01	0.99 (0.80, 1.21)	0.90
High	1441	0.51 (0.42, 0.62)	<0.01	0.84 (0.67, 1.07)	0.16

Abbreviations: OR, Odds Ratio; 95% CI, 95% Confidence Interval; 25(OH)D, 25-hydroxyvitamin D; LDL-cholesterol, low-density lipoprotein cholesterol; SD, standard deviation; IQR, interquartile range. ^§^ Multivariable odds ratios are adjusted for all covariates in the table. **^†^** Elevated LDL-cholesterol was defined as LDL-cholesterol concentration ≥2.6 mmol/L. **^‡^** Blood pressure status was defined based on blood pressure ≥140/90 mm Hg, or a self-report of taking antihypertensive medications as “elevated”.
